# Resveratrol Inhibits the TGF-*β*1-Induced Proliferation of Cardiac Fibroblasts and Collagen Secretion by Downregulating miR-17 in Rat

**DOI:** 10.1155/2018/8730593

**Published:** 2018-12-17

**Authors:** Yao Zhang, Yuan Lu, Machuki Jeremiah Ong'achwa, Liqi Ge, Yun Qian, Lei Chen, Xiaoqin Hu, Fei Li, Hui Wei, Chaoqun Zhang, Chengzong Li, Zhirong Wang

**Affiliations:** Department of Cardiology, Affiliated Hospital of Xuzhou Medical University, Xuzhou 221002, China

## Abstract

Myocardial fibrosis (MF) can cause heart remodeling and it is an independent risk factor for malignant arrhythmias, sudden cardiac death, and other malignant cardiovascular events. It is often characterized by myocardial interstitial collagen deposition and hyperproliferation of cardiac fibroblasts (CFs). The transforming growth factor-*β*1 (TGF-*β*1) is the most influential profibrogenic factor. Resveratrol (RSV) is an active polyphenol substance that inhibits myocardial fibrosis. The mechanism of RSV-mediated inhibition of the proliferation of CFs at the microRNA level is not fully understood. We used TGF-*β*1 to induce CFs proliferation to simulate the pathogenesis of myocardial fibrosis. Neonatal rat CFs were treated with TGF-*β*1 in the presence or absence of resveratrol. Cell proliferation was measured using the CCK-8 and EdU assay. Collagen secretion was measured using hydroxyproline kit. Further, qPCR analysis was performed to determine microRNA levels after TGF-*β*1 or resveratrol treatment. To identify the target gene for miR-17, miR-17 was overexpressed or silenced, and the mRNA and protein levels of Smad7 were assessed. The effects of miR-17 silencing or Smad7 overexpression on cell proliferation and collagen secretion were also examined. Resveratrol treatment significantly decreased the TGF-*β*1-induced CF proliferation and collagen secretion. Resveratrol also decreased the levels of miR-17, miR-34a, and miR-181a in TGF-*β*1-treated CFs. Overexpression of miR-17 decreased the Smad7 mRNA and protein levels while silencing miR-17 increased them. Additionally, silencing miR-17 or overexpressing Smad7 decreased the TGF-*β*1-induced CFs proliferation and collagen secretion. In conclusion, resveratrol inhibits TGF-*β*1-induced CFs proliferation and collagen secretion. This inhibitory effect of resveratrol is orchestrated by the downregulation of miR-17 and the regulation of Smad7.

## 1. Introduction

Myocardial fibrosis (MF) refers to the excessive deposition of collagen fibers, increased collagen content, or altered collagen composition in heart tissue. MF affects systolic and diastolic function [1]. In addition, MF is an independent risk factor for malignant arrhythmias, sudden cardiac death, and many other malignant cardiovascular events [2]. It is often characterized by myocardial interstitial collagen deposition and the hyperproliferation of cardiac fibroblasts (CFs) [3, 4]. CFs are the most abundant cell-type in the heart, which play a key role in myocardial fibrosis as well as heart remodeling [5]. Therefore, it is important to study the pathomechanism of MF and develop drugs for the treatment of myocardial fibrosis in patients. The transforming growth factor-*β*1 (TGF-*β*1), a key factor in MF [6], is the most important profibrogenic factor and it can induce the proliferation, collagen secretion, and transdifferentiation of CFs via autocrine or paracrine pathways. TGF-*β*1 acts through the Smad signaling pathway to induce fibrosis. Smad proteins are the only molecules that transduce the TGF-*β*1 signals from the cell membrane to the nucleus [7]. Once TGF-*β*1 binds to its receptors, type II/type I TGF-beta receptors, they become activated which in turn activate other cellular proteins. The downstream target molecules of these receptors include Smad2, Smad3, and Smad4. These proteins interact to form a common complex or a heterodimer, which translocates to the nucleus and binds to specific DNA sequences. These conjugating proteins initiate the transcription of their target genes, thereby affecting their biological effects in the target cells. Smad6 and Smad7 are negative regulators of this signaling pathway [8]. Smad7 inhibits the phosphorylation of Smad2, prevents the interaction between Smad2 and Smad4, and prevents the Smad complex from translocating to the nucleus. Therefore, it is possible that Smad7 may negatively regulate the TGF-*β*1/Smad signaling pathway. Inhibition of TGF-*β*1-induced CFs proliferation and collagen secretion may inhibit myocardial fibrosis and reduce cardiovascular disease mortality.

MicroRNAs (miRNAs, miRs) are endogenous noncoding RNAs with lengths of approximately 22 nucleotides. MicroRNAs are highly conserved and widely distributed in many animals, plants, and microorganisms. The first microRNA, lin-4, was discovered by Lee et al. in 1993 in* Caenorhabditis elegans* [9]. MicroRNAs act posttranscriptionally on mRNAs by binding to their 3' untranslated regions (3' UTRs), thereby preventing the translation of mRNAs or causing their degradation. They are involved in the regulation of proliferation, apoptosis, metabolism, invasion, and other physiological or pathological processes [10–12]. It has also been reported that microRNAs play a role in the regulation of myocardial fibrosis. For instance, MicroRNA-208 is upregulated in the myocardial tissue after myocardial infarction, where it inhibits the THRAP1 and regulates postmyocardial fibrosis [13]. miR-21 regulates the ERK-MAP kinase signaling pathway and promotes the CFs proliferation and collagen secretion by inhibiting the expression of spryl [14]. In other organs, microRNAs also play important roles in regulating fibrosis. For example, miR-17 has been reported to inhibit Smad7 and to promote the proliferation and transdifferentiation of hepatic stellate cells and collagen secretion [15]. miR-181a can promote AKT phosphorylation by targeting PHLPP2, which promotes the proliferation and inhibits the apoptosis of keloid fibroblasts [16].

Resveratrol (RSV) is an active polyphenol substance that is found in grapes and knotweed. It has antibacterial, anti-inflammatory, and immunoregulatory effects [17, 18]. In the cardiovascular system, it reduces ischemia-reperfusion injury and arrhythmias [19, 20]. Additionally, Wang et al. reported that RSV inhibited the angiotensin II-induced proliferation of CFs by activating the NO-cGMP pathway [21]. Moreover, Olson et al. found that RSV inhibited the proliferation and transdifferentiation of CFs by suppressing the ERK1/2 signaling cascade [22].

However, the mechanism of RSV-mediated inhibition of the proliferation of CFs at the microRNA level is not fully understood. In this study, we used TGF-*β*1 to induce CFs proliferation to simulate the pathogenesis of myocardial fibrosis and tested the effect of RSV treatment on the proliferation of CFs and collagen secretion induced by TGF-*β*1. We selected miR-17 [15], which regulates the proliferation of hepatic stellate cells; miR-34a [23], which regulates the proliferation of CFs; and miR-181a, which regulates the proliferation of keloid fibroblasts [16] to investigate whether the levels of these microRNAs in CFs were influenced by TGF-*β*1 and RSV treatment. Then miR-17 was chosen for further exploration of the mechanism involved. This study may provide new experimental evidence for the therapeutic effects of RSV for the treatment of myocardial fibrosis using.

## 2. Materials and Methods

### 2.1. Animals and Reagents

This study was approved by the Animal Ethics Committee of Xuzhou Medical University. SD rats (1-3 days old) were purchased from the Laboratory Animal Center (Xuzhou Medical University). The animals used in this study received humane care and handling. RSV and type I collagenase were obtained from Sigma-Aldrich (St. Louis, MO, USA). Recombinant human TGF-*β*1 was purchased from Peprotech (Suzhou, China). Dulbecco's modified Eagle's medium (DMEM) and fetal bovine serum (FBS) were purchased from Life Technologies (Grand Island, NY, USA). Cell Counting Kit-8 (CCK-8), 0.25% trypsin, dimethyl sulfoxide (DMSO), RIPA lysis buffer, phenylmethylsulfonyl fluoride (PMSF), and the bicinchoninic acid (BCA) protein assay kit were purchased from Beyotime (Guangzhou, China). The EdU (5-ethynyl-2-deoxyuridine) staining kit was purchased from RiboBio (Guangzhou, China). The hydroxyproline assay kit was purchased from Jiancheng Bioengineering Institute (Nanjing, China). A polymerase chain reaction (PCR) kit was purchased from Tiangen (Beijing, China). The mouse monoclonal antibody against Smad7 was purchased from Santa Cruz Biotechnology, Inc. (Paso Robles, CA, USA). The rabbit polyclonal antibody against GAPDH was purchased from Proteintech Group, Inc. (Rosemont, IL 60018, USA). Secondary antibodies conjugated to HRP were purchased from Zhongshan Jinqiao Biotechnology (Beijing, China).

### 2.2. Cell Culture

Hearts were isolated from neonatal SD rats (1-3 days old) and washed in cold PBS. Then, they were transferred into a serum bottle and cut into pieces. The tissues were digested with equal volumes of 0.08% trypsin and 0.08% collagenase I at 37°C for 5 min per digest on a magnetic stirrer. The first supernatant was discarded, while the supernatant of each subsequent digest was collected in DMEM containing 10% FBS to terminate the reaction. The digestion was complete when the supernatant was clear. Thereafter, the cell suspension was collected and centrifuged at 1000 rpm for 5 min. The supernatant was discarded and the precipitate was resuspended. The new cell suspension was transferred onto a Petri dish and then incubated in 95% O_2_ and 5% CO_2_ at 37°C. After 1-1.5 h, the CFs were adhered according to the differential adhesion time. After 2-3 days, the cells were digested with 0.25% trypsin when they grew to 85-90% confluence and were passaged at a 1:2 ratio. The second and third generations of the cells were used for subsequent experiments.

### 2.3. Cell Viability Assay

RSV was dissolved in sterile DMSO to prepare a stock concentration of 3.0×10^4^ *μ*M. The RSV solution was further diluted with DMEM medium to obtain different working concentrations before use. The final concentration of DMSO was less than 0.01%. The second and third generations of fibroblasts were seeded in 96-well plates at a density of 10^4^ cells per well for 24 h. After starvation, the cells were treated with RSV at 12.5*μ*M, 25*μ*M, 50*μ*M, 75*μ*M, and 100*μ*M for 12 h, 24 h, and 48 h. The cells in the control group were incubated with normal medium without RSV, and those of the DMSO group were exposed to 0.01% DMSO in the medium. Each group was set up in 5 replicates. Ten microliters of CCK-8 solution were added to each well, and the cells were incubated in an incubator with 5% CO_2_ at 37°C for 4 h. The absorbance of the cells at 450 nm was measured with a spectrophotometer (Thermo Fisher Scientific, Waltham, MA, USA).

### 2.4. Cell Proliferation Assay

Regarding EdU incorporation assay, after the CFs were exposed to different treatments, 50*μ*M EdU was added to the medium and incubated for 2 h. Then the cells were fixed with 4% paraformaldehyde for 30 min at room temperature. 1×Apollo® Staining Solution (red) was added to each well and the cells were incubated for 30 min at room temperature. In addition, Hoechst 33342 solution was added to each well, and the cells were incubated for additional 5 min. Staining results were analyzed using a microscope (Olympus, Tokyo, Japan), and the number of positive cells was determined in 5 random views.

For CCK-8 assay, CFs were seeded on 96-well culture plates for 24 h. After an overnight starvation by serum deprivation, CFs were incubated for 48 h in a medium containing 5ng/mL TGF-*β*1 plus 0.01% DMSO, 5ng/mL TGF-*β*1 plus 50*μ*M RSV, or 0.01% DMSO as the control. Thereafter, the CFs were cultured in a normal culture medium containing 10% CCK-8 at 37°C for 4 h. The absorbance was determined at 450 nm using a spectrophotometer (Thermo Fisher Scientific, Waltham, MA, USA).

### 2.5. Collagen (Hydroxyproline) Content Assay

The hydroxyproline contents in the cell lysis buffer and supernatants were determined with the hydroxyproline assay kit (Jiancheng, Nanjing, China) according to the manufacturer's instructions [24]. Absorbance was detected at 550 nm using a microplate reader (Bio-Rad550, California, USA).

### 2.6. mir-17 Mimic/Inhibitor and Smad7 Overexpression

A mir-17 mimic/inhibitor and negative control were purchased from GenePharma (Suzhou, China). CFs were cultured in 6-well culture plates to reach 60% confluence. Subsequently, the cells were transfected with miR-17 mimic/inhibitor using Lipofectamine 2000 (Invitrogen, Carlsbad, CA, USA) according to the manufacturer's instructions.

The Smad7 expression vector named as pcDNA3.1(+)-Smad7 plasmid was constructed by GenePharma (Suzhou, China). CFs seeded on 6-well culture plates at a 60% confluence were transfected with pcDNA3.1(+)-Smad7/pcDNA3.1(+)-NC using Lipofectamine 2000 (Invitrogen, Carlsbad, CA, USA) according to the manufacturer's manual.

After transfection, each group was treated with TGF-*β*1 or RSV as indicated for 48 h.

### 2.7. RNA Isolation and Reverse Transcription-Quantitative Polymerase Chain Reaction (RT-qPCR)

Total RNA was extracted with TRIZOL (Invitrogen, Carlsbad, CA, USA) according to the manufacturer's instructions. 2*μ*g RNA was reverse-transcribed to cDNA by stem-loop methods with the TIANScript RT Kit (Tiangen, Beijing, China). SuperReal PreMix Plus (SYBR Green) (Tiangen, Beijing, China) was used to detect the expression of the microRNAs, and the 5S was used as the internal reference. The stem-loop, forward primers, and the universal reverse primer were designed based on the sequences of rat miR-17, miR-181a, miR-34a, and 5S. The primer sequences are shown in Table 1. For RT-qPCR analysis of Smad7, the total RNA was reverse-transcribed to cDNA using the TIANScript RT Kit (Tiangen, China). The PCR assay was implemented using SuperReal PreMix Plus (SYBR Green) (Tiangen, China), and GAPDH was used as the endogenous control gene. The primer sequences of Smad7 were as follows: forward, 5'-GGAGTCCTTTCCTCTCTC-3'; reverse, 5'-GGCTCAATGAGCATGCTTCAC-3'. The GAPDH primer sequences were as follows: forward, 5'-TCTCTGCTCCTCCCTGTTC-3'; reverse, 5'-ACACCGACCTTCACCATCT-3'. The primers were synthesized by Generay Company (Shanghai, China). PCR was performed on ABI7500 Real-Time PCR appliance. The cycling conditions were as follows: 95°C for 15 min, 95°C for 10 sec, and 60°C for 32 sec. The relative quantitative analysis of the expression was calculated using the 2^−ΔΔCt^ method.

### 2.8. Western Blot

CFs were harvested and lysed in cold RIPA lysis buffer containing phenylmethylsulfonyl fluoride (PMSF) for 30 min and then centrifuged at 12,000×g for 15 min at 4°C. The supernatants were collected and the protein concentration was measured using the bicinchoninic acid (BCA) protein assay kit (Beyotime, Guangzhou, China). Total protein from each sample was separated by sodium dodecyl sulfate polyacrylamide gel electrophoresis, and the proteins were transferred to polyvinylidene fluoride membranes. After blocking with 5% nonfat dry milk in TBS for 2 h at room temperature, the membranes were incubated with Smad7 (1: 1000, Santa Cruz) or GAPDH (1: 1000, Proteintech Group) overnight at 4°C and then probed with secondary antibodies [HRP-labelled goat anti-rabbit IgG (H+L) and HRP-labelled goat anti-mouse IgG (H+L) (1: 10,000; Zhongshan Jinqiao Biotechnology, Beijing, China)] for 1 h at room temperature. Visualization of the protein bands was performed using an enhanced chemiluminescence (ECL) method. The densities of the bands were normalized to those of the corresponding GAPDH bands and then normalized to those of the control group, which was set to a value of 1.

### 2.9. Statistical Analysis

Data are expressed as the mean ± SD ($\bar{x}\pm s$). Statistical analysis was performed with SPSS 16.0 (Chicago, USA). Differences between multiple groups were analyzed using one-way analysis of variance (ANOVA), followed by Newman-Keuls comparison tests. P≤0.05 was considered statistically significant. GraphPad Prism 5 (GraphPad Prism, San Diego, CA, USA) was used to generate data graphs.

## 3. Results

### 3.1. Optimal Concentration and Time for RSV Treatment

**Figure 1:**
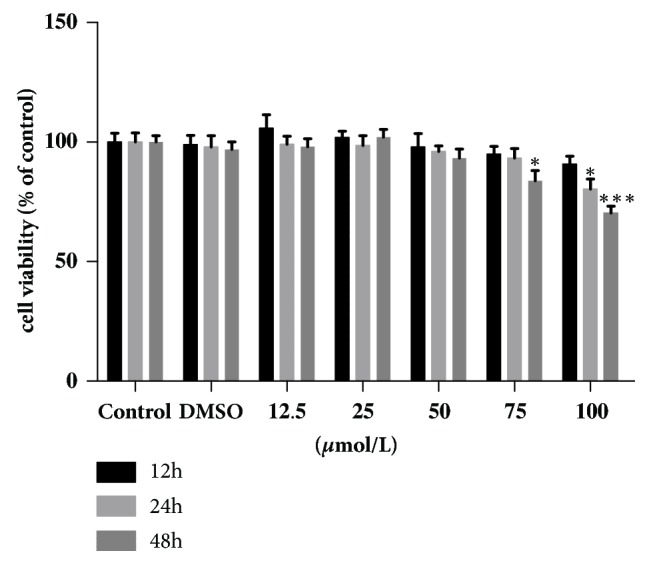
Cell viability of CFs treated with resveratrol at different concentrations for different durations. *∗∗*P<0.01 and *∗∗∗*P<0.001 versus the control group (n=5).

The CCK-8 assay was used to measure viability of the CF. As shown in Figure 1, there was no significant difference in CF viability in the RSV groups treated with 12.5*μ*M to 100*μ*M RSV for 12 h when compared with the control group and DMSO group. In addition, there was no significant difference in CF viability between the control group and DMSO group. After 24 h, cell viability was obviously decreased in the group treated with 100*μ*M (P<0.05), which was 80.33% of the control group. After 48 h, cell viability decreased to 83.84% in 75*μ*M RSV group and to 70.35% in 100*μ*M RSV group. Our aim was to determine the highest concentration and the longest duration that does not affect cell viability. Therefore, 50*μ*M RSV was used in the subsequent experiments to treat CFs for 48 h (Figure 1).

### 3.2. Resveratrol Inhibits TGF-*β*1-Induced CF Proliferation

**Figure 2:**
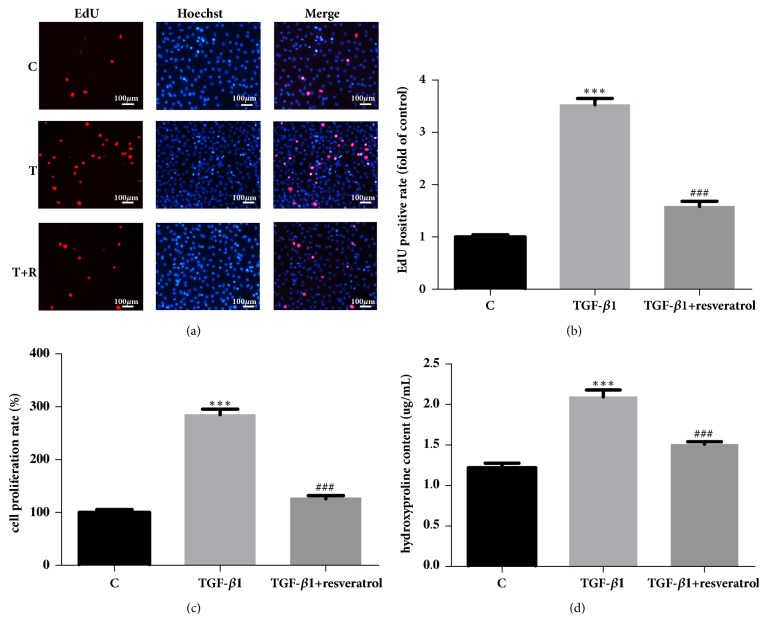
The effect of resveratrol on TGF-*β*1-induced cardiac fibroblast proliferation and collagen secretion. (a) Representative images. All cell nuclei were stained with Hoechst33342 (blue). The proliferating cell nuclei were stained with 1×Apollo® Staining Solution (red). C: control; T: TGF-*β*1; T+R: TGF-*β*1+RSV. (b) The relative ratio of EdU-positive cells. (c) The CF proliferation rate as assessed by CCK-8 assay. (d) The secretion of hydroxyproline as measured using the hydroxyproline assay kit. *∗∗∗*P<0.001 versus the control group and ###P<0.001 versus the TGF-*β*1 group (n=3).

The EdU assay revealed that the number of EdU-positive cells increased significantly in the TGF-*β*1 group compared with the control group. After RSV treatment, the number of EdU-positive cells decreased markedly compared to the TGF-*β*1 group (Figures 2(a) and 2(b)). The result of the CCK-8 assay was similar to that of the EdU assay (Figure 2(c)).

### 3.3. Resveratrol Inhibits TGF-*β*1-Induced CF Collagen Secretion (Hydroxyproline)

Hydroxyproline accounts for approximately 13.4% of collagen and its quantity in elastin is very low. Hydroxyproline is not present in other proteins. Therefore, the hydroxyproline content in the cell supernatant can be used to reflect the amount of collagen secreted by CFs. The oxidation products of hydroxyproline produced by actions of oxidants and dimethylaminobenzaldehyde show a purple-red color. Hence, the hydroxyproline content can be inferred by the color intensity. In this experiment, the OD value was measured with a microplate reader and the hydroxyproline content was calculated using the formula described in Materials and Methods. The hydroxyproline assay kit showed that, compared with the control group, treatment with 5ng/mL TGF-*β*1 for 48 h promoted the secretion of collagen by CFs significantly (P<0.001). Furthermore, the addition of 50*μ*M RSV significantly inhibited the TGF-*β*1-induced collagen secretion by the CFs (P<0.001) (Figure 2(d)).

### 3.4. Effects of RSV on the Expression of miR-17, miR-34a, and miR-181a in TGF-*β*1-Treated CFs

**Figure 3:**
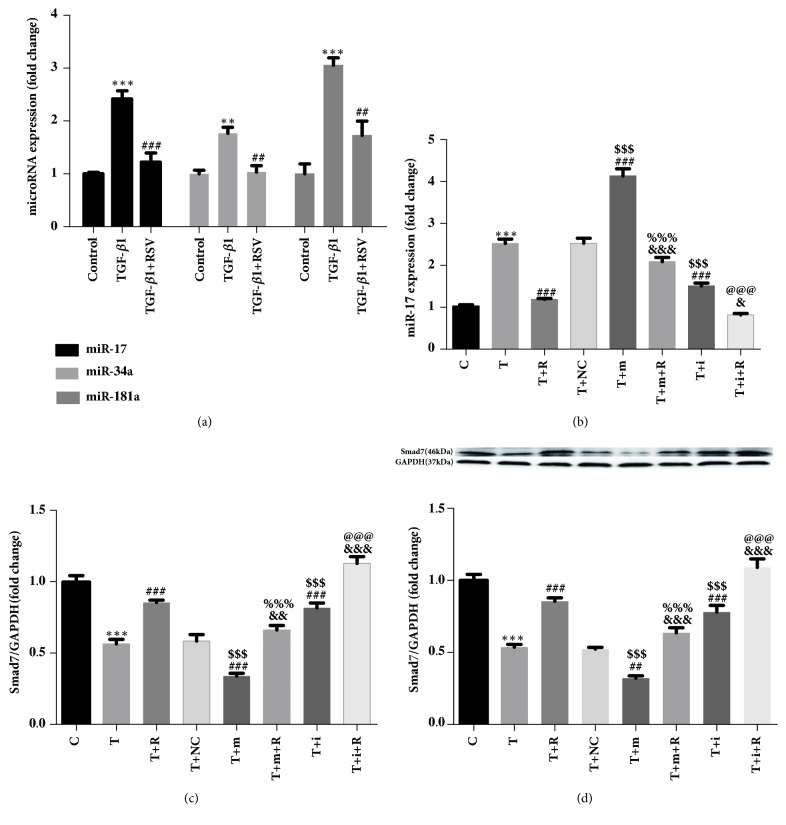
Effect of resveratrol on miRNAs and Smad7 mRNA/protein expression in CFs. (a) Analysis of differential miRNA expression in CFs by qPCR. (b) miR-17 expression in CFs after miR-17 overexpression/silencing. (c) Smad7 mRNA expression in CFs after miR-17 overexpression/silencing. (d) Smad7 protein expression in CFs after miR-17 overexpression/silencing. *∗∗∗*P<0.001 versus the control group; ###P<0.001 versus the TGF-*β*1 group; &&&P<0.001 and &P<0.05 versus the TGF-*β*1+resveratrol group; $$$P<0.001 versus the TGF-*β*1+NC group; %%%P<0.001 versus the TGF-*β*1+miR-17 mimics group; @@@P<0.001 versus the TGF-*β*1+ miR-17 inhibitor group (n=3). C: Control; T: TGF-*β*1; T+R: TGF-*β*1+RSV; T+NC: TGF-*β*1+negative control; T+m: TGF-*β*1+miR-17 mimics; T+i: TGF-*β*1+miR-17 inhibitor; T+m+R: TGF-*β*1+miR-17 mimics+RSV; T+i+R: TGF-*β*1+miR-17 inhibitor+RSV.

The expression levels of miR-17, miR-34a, and miR-181a were upregulated in the TGF-*β*1 group compared with those in the control group. The expression levels of miR-17, miR-34a, and miR-181a in the TGF-*β*1+RSV group were downregulated compared with those of the TGF-*β*1 group (Figure 3(a)).

### 3.5. Effects of Resveratrol on miR-17 Expression in CFs after miR-17 Overexpression/Silencing

We used qPCR to measure the miR-17 expression in CFs after miR-17 overexpression/silencing. The results showed that the miR-17 expression was higher in the TGF-*β*1 group compared with the control group, and its expression in the TGF-*β*1+RSV group was lower compared with the TGF-*β*1 group. There was no significant difference in miR-17 expression between the TGF-*β*1 group and TGF-*β*1+negative control group. Expression of miR-17 in the TGF-*β*1+miR-17 mimics group was higher compared with the TGF-*β*1+negative control group, and its expression in the TGF-*β*1+miR-17 inhibitor group was decreased. The expression of miR-17 in the TGF-*β*1+miR-17 mimics+RSV group was lower than that of the TGF-*β*1+miR-17 mimics group. Compared with the TGF-*β*1+miR-17 inhibitor group, the expression of miR-17 in the TGF-*β*1+miR-17 inhibitor+RSV group was decreased (Figure 3(b)).

### 3.6. Effect of Resveratrol on mRNA and Protein Expression of Smad7 in CFs after miR-17 Overexpression/Silencing

After miR-17 overexpression/silencing, the results obtained from qPCR showed that the expression of Smad7 mRNA in the TGF-*β*1 group was lower compared with the control group, and its expression in the TGF-*β*1+RSV group was higher than that of TGF-*β*1 group. There was no significant difference in Smad7 mRNA expression between the TGF-*β*1 group and TGF-*β*1+negative control group. Compared with the TGF-*β*1+negative control group, the expression of Smad7 mRNA in the TGF-*β*1+miR-17 mimics group was lower, while its expression in the TGF-*β*1+miR-17 inhibitor group was increased. The expression of Smad7 mRNA in the TGF-*β*1+miR-17 mimics+RSV group was higher than that of TGF-*β*1+miR-17 mimics group. Compared with the TGF-*β*1+miR-17 inhibitor group, the expression of Smad7 mRNA in the TGF-*β*1+miR-17 inhibitor+RSV group was increased (Figure 3(c)). The expression trend for Smad7 protein was similar to that of its mRNA (Figure 3(d)).

### 3.7. Effects of miR-17 and Smad7 on the TGF-*β*1-Induced Proliferation of CFs and Collagen Secretion

**Figure 4:**
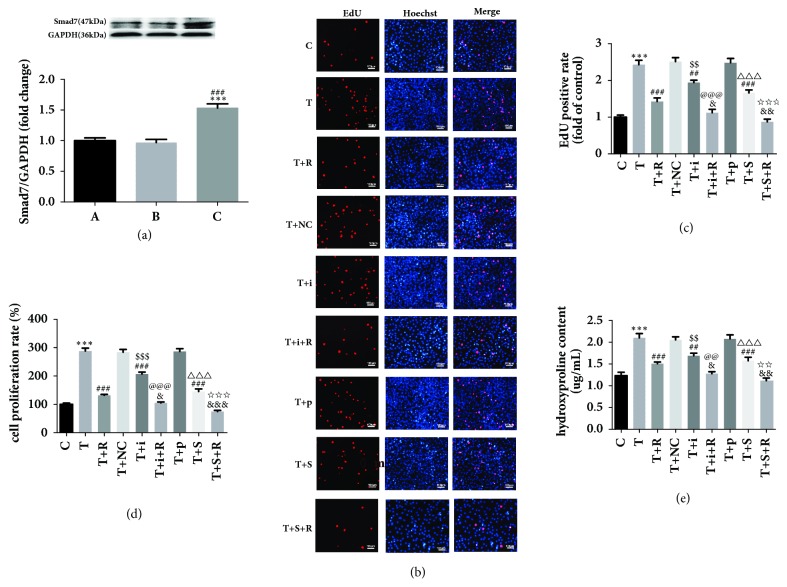
The effect of miR-17 and Smad7 on the proliferation of cardiac fibroblasts and collagen secretion after TGF-*β*1 treatment. (a) Effect of transfection of Smad7 overexpression plasmid on Smad7 protein levels in each group. A: control; B: pcDNA 3.1(+) vector; C: pcDNA 3.1(+)-Smad7. (b) Representative images of EdU incorporation assays. C: control; T: TGF-*β*1; T+R: TGF-*β*1+RSV; T+NC: TGF-*β*1+NC; T+i: TGF-*β*1+miR-17 inhibitor; T+i+R: TGF-*β*1+miR-17 inhibitor+RSV; T+p: TGF-*β*1+pcDNA.3.1(+) vector; T+S: TGF-*β*1+pcDNA.3.1(+)-Smad7; T+S+R: TGF-*β*1+pcDNA.3.1(+)-Smad7+RSV. (c) The CF proliferation rate as determined by CCK-8 assay. (d) The relative ratio of EdU-positive cells. (e) The secretion of hydroxyproline as measured using the hydroxyproline assay kit. *∗∗∗*P<0.001 versus the control group; %%%P<0.001 versus the pcDNA.3.1(+) vector group; ##P<0.01 and ###P<0.001 versus the TGF-*β*1 group; &P<0.05, &&P<0.01, and &&&P<0.001 versus the TGF-*β*1+RSV group; $$P<0.01 and $$$P<0.001 versus the TGF-*β*1+NC group; @@P<0.01 and @@@P<0.001 versus the TGF-*β*1+miR-17 inhibitor group; △△△P<0.001 versus the TGF-*β*1+pcDNA.3.1(+) vector group; ☆☆P<0.01 and ☆☆☆P<0.001 versus the TGF-*β*1+pcDNA.3.1(+)-Smad7 group (n=3).

The expression of Smad7 protein in CFs was detected by Western blot. The results showed that there was no significant difference in the expression of Smad7 protein in the pcDNA.3.1 (+) vector group compared with the control group. Protein expression of Smad7 in the pcDNA.3.1 (+)-Smad7 group was significantly higher compared with that of pcDNA.3.1 (+) vector group. This shows that the transfection overexpression plasmid used in this experiment succeeded in overexpressing Smad7 protein (Figure 4(a)).

EdU assays were performed to measure CF proliferation after miR-17 silencing or Smad7 overexpression. The results showed that there was no significant difference in the number of EdU-positive cells between the TGF-*β*1 group and TGF-*β*1+negative control group. CF proliferation in the TGF-*β*1+miR-17 inhibitor group was lower compared with that of TGF-*β*1 or TGF-*β*1+NC group. On the other hand, the CF proliferation in TGF-*β*1+RSV+miR-17 inhibitor group was lower compared with that of the TGF-*β*1+RSV group. Moreover, CF proliferation in the TGF-*β*1+pcDNA.3.1 (+)-Smad7 group was lower than that of TGF-*β*1 or TGF-*β*1+ pcDNA.3.1 (+) vector group. CF proliferation in the TGF-*β*1+RSV+pcDNA.3.1 (+)-Smad7 group was lower compared with the TGF-*β*1+RSV group (Figures 4(b) and 4(c)). Results from the CCK-8 and hydroxyproline assays were similar to those described above (Figures 4(d) and 4(e)).

## 4. Discussion

Myocardial fibrosis refers to an increase in composition of the extracellular matrix (ECM) due to high collagen secretion and deposition in myocardial tissue leading to abnormal remodeling of the entire interstitial network of the heart [25]. Myocardial fibrosis is associated with multiple cardiovascular diseases. Abnormally increased and disorganized collagen architecture causes the atrial myocytes to be segregated which impairs intercellular electrical conduction leading to atrial fibrillation [26]. In addition, myocardial fibrosis is closely related to cardiomyopathy, myocardial infarction, and other cardiovascular diseases. In fact, many cardiovascular diseases are thought to develop myocardial fibrosis in the final stage [27]. Ventricular remodeling is an important feature of myocardial fibrosis and it is characterized by decreased cardiac compliance, abnormal diastolic and systolic function of the heart, malignant arrhythmias, and sudden cardiac death. Myocardial fibrosis occurs due to increased proliferation, transdifferentiation, and collagen secretion by CFs. Inhibition of CF proliferation and collagen secretion are important in preventing and treating myocardial fibrosis. TGF-*β*1 is a key factor in the development of myocardial fibrosis; it can induce CF proliferation and transdifferentiation. In addition, TGF-*β*1 can promote the deposition of ECM and it plays an important role in heart remodeling. Downstream pathways of TGF-*β*1 are categorized as classical T*β*R1-Smad2/3 [28] and a nonclassical TAK1/p38 pathway [29]. It has been shown that TGF-*β*1 promotes the expression of type I collagen through the ROCK/MRTA pathway and stimulates the production of ET1, thereby activating ECM synthesis and transdifferentiation of fibroblasts into myofibroblasts via JNK pathway activation [30].

RSV is a nonflavonoid polyphenol that is reported to possess antitumor effects, such as promoting apoptosis of human breast cancer cells [31] and inhibiting invasion and migration of pancreatic cancer cells [32]. Additionally, it can inhibit apoptosis, reduce the myocardial infarct size, and protect the cardiovascular system [19, 33]. It also possesses antifibrosis effects. Olson et al. reported that RSV can inhibit CF proliferation by attenuating the EGF-activated ERK1/2 signaling cascade [22]. Furthermore, RSV can inhibit the angiotensin II-induced proliferation of human lung fibroblasts in vitro by activating the NO-cGMP pathway [21].

MicroRNAs are endogenous noncoding RNAs with approximately 22 nucleotides. MicroRNAs can bind to the 3' UTRs of target mRNAs, thereby preventing the translation of target mRNAs or causing their degradation. MicroRNAs play important roles in the development of fibrosis in various organs and tissues. In myocardial tissues, miR-21 targets endogenous mitogen activated protease inhibitor Spry1 and activates the ERK-MAPK pathway, thus inhibiting apoptosis of CFs and activating myocardial fibrosis [34]. In canine atrial fibroblasts, miR-29 knockout increased the expression of COL1A1, COL3A1, and FBN significantly [35]. miR-34a promoted TGF-*β*1-induced CF proliferation and transdifferentiation and collagen secretion by regulating Smad4 [23]. In hepatic tissues, miR-17 promoted the proliferation of hepatic stellate cells, leading to hepatic fibrosis by inhibiting Smad7 [15]. Moreover, miR-181a targeted at PHLPP2 enhances AKT phosphorylation, which activates the proliferation of keloid fibroblasts and inhibits apoptosis [16].

Currently, there are few studies on the mechanism of RSV-induced inhibition of CFs proliferation and collagen secretion at the microRNA level. In this study, TGF-*β*1 was used to induce CFs proliferation and collagen secretion to model the pathogenesis of myocardial fibrosis. Thereafter, the inhibitory effect of RSV on myocardial fibrosis model was studied. Firstly, we used the CCK-8 and EdU proliferation assay kits to measure CF proliferation in the control group, TGF-*β*1 group, and TGF-*β*1+RSV group. The results showed that TGF-*β*1-induced CF proliferation significantly, which was inhibited by RSV. The hydroxyproline kit was used to measure collagen secretion in all groups; the results showed that the TGF-*β*1-induced collagen secretion by CFs was inhibited by RSV.

We performed qPCR to measure the microRNAs associated with proliferation [15, 16, 23]. The results showed that, when treated with TGF-*β*1, the levels of miR-17, miR-34a, and miR-181a in CFs were increased, but after treatment with RSV, the levels of these microRNAs were decreased. Further, we selected miR-17 to study the mechanism of RSV inhibition of TGF-*β*1-treated CFs. Smad7 gene is among the target genes of miR-17 that were predicted by the miRDB software and is associated with the TGF-*β*1/Smad signaling pathway [36, 37]. Luciferase reporter assays have revealed that rat Smad7 mRNA is the target gene of rno-miR-17 [15]. Therefore, we used PCR and Western blot methods to evaluate the expression levels of Smad7 mRNA and protein in each group after overexpressing/silencing miR-17. The PCR results showed that, after miR-17 was overexpressed, Smad7 mRNA expression was significantly downregulated in the TGF-*β*1+miR-17 mimics group compared with the TGF-*β*1 group and TGF-*β*1+NC group. After RSV treatment, expression of Smad7 mRNA was significantly upregulated. Conversely, silencing miR-17 significantly upregulated the expression of Smad7 mRNA in the TGF-*β*1+miR-17 inhibitor group compared with the TGF-*β*1 group and TGF-*β*1+NC group. These findings confirm that miR-17 can regulate Smad7 mRNA in TGF-*β*1-induced CFs. The results of the Western blot analysis were similar to those of the PCR analysis, which further verified that miR-17 regulates the expression of Smad7 protein in TGF-*β*1-induced CFs.

We further examined the effects of miR-17 and Smad7 on the proliferation of CFs and collagen secretion. We used an inhibitor to silence miR-17 in CFs. The CCK-8 and EdU results showed that CF proliferation in the TGF-*β*1+miR-17 inhibitor group was remarkably suppressed after miR-17 silencing compared with CF proliferation in the TGF-*β*1 group or TGF-*β*1+NC group. After RSV treatment, cell proliferation was decreased remarkably. Therefore, miR-17 silencing can inhibit the TGF-*β*1-induced CF cell proliferation. In TGF-*β*1-treated CFs, overexpression of Smad7 suppressed cell proliferation and RSV treatment further weakened it. Similarly, the hydroxyproline assay kit results indicated that the miR-17 inhibitor suppressed the TGF-*β*1-induced CF collagen secretion and that Smad7 overexpression suppressed the TGF-*β*1-induced CF collagen secretion.

In conclusion, this study shows that RSV has an inhibitory effect on TGF-*β*1-induced CF proliferation and collagen secretion, which may be related to the downregulation of miR-17 and subsequent regulation of Smad7 mRNA and protein expression. These results provide a new theoretical basis and experimental support of the clinical application of RSV for the treatment of myocardial fibrosis.

## 5. Conclusions

We demonstrate that resveratrol inhibits TGF-*β*1-induced CFs proliferation and collagen secretion. This inhibitory effect of resveratrol is orchestrated by the downregulation of miR-17 and the regulation of Smad7. This study provides new experimental evidence for the therapeutic effects of RSV for the treatment of myocardial fibrosis using.

## Figures and Tables

**Table 1:** Primer sequences of 5S and miRNAs for real time-polymerase chain reaction.

Gene	primer	Sequence
5S	Forward	TGGGTTCATTTCTGGGTCTT
5S	Reverse	GGATGGGAGACCGCCTGGGAATAC
5S	Stem-loop	GAGTAGACCAATGGGTTCATTTCTGGGTCTTATTCTATTCCATTGGTCTACTCAAAGCCTA
miR-17	Forward	GTACCAAAGTGCTTACAGTGC
miR-17	Stem-loop	GAGTAGACCAATGGGTTCATTTCTGGGTCTTATTCTATTCCATTGGTCTACTCCTACCT
miR-181a	Forward	GTCTAACATTCAACGCTGTCG
miR-181a	Stem-loop	GAGTAGACCAATGGGTTCATTTCTGGGTCTTATTCTATTCCATTGGTCTACTCACTCAC
miR-34a	Forward	GTCTTGGCAGTGTCTTAGCT
miR-34a	Stem-loop	GAGTAGACCAATGGGTTCATTTCTGGGTCTTATTCTATTCCATTGGTCTACTCACAACC

The reverse primers of all microRNAs were the same as those of 5S.

## Data Availability

The data used to support the findings of this study are available from the corresponding author upon request.
